# Multi-Target Bioactivity of *Dittrichia viscosa* Polyphenols: HPLC-ESI-MS Profiling, Antimicrobial Mechanisms, and Molecular Docking

**DOI:** 10.3390/ph19081139

**Published:** 2026-07-23

**Authors:** Bahia Abdelfattah, Oussama Khibech, Amena Mrabet, Jaber Maataoui, Amr Kchikich, Widad Stitou, Ayoub Simou, Abdelaaty A. Shahat, Joe Miantezila Basilua, Rashed N. Herqash, Asmae El Cadi, Mohamed Khaddor

**Affiliations:** 1 Laboratory of Physical Chemistry of Materials, Natural Substances and Environment (LAMSE), Chemistry Department, Faculty of Sciences and Techniques of Tangier, Abdelmalek Essaadi University, Tangier 90000, Morocco; 2 Laboratory of Applied Chemistry and Environment (LCAE), Faculty of Science of Oujda, Mohammed First University, Bd Mohamed VI BP 717, Oujda 60000, Morocco; 3 National Sheep and Goat Association (ANOC), Mers El Kheir 12025, Morocco; amr.kchikich@etu.uae.ac.ma; 4 Center for Research on Medicinal, Aromatic, and Poisonous Plants, DSR, King Saud University, Riyadh P.O. Box 2457, Saudi Arabia; 5 Department of Biostatistics and Mathematics, Faculty of Pharmacy, Paris Cité University, 4, Avenue de L’observatoire, 75006 Paris, France

**Keywords:** antimicrobial activity, antioxidant activity, *Dittrichia viscosa*, HPLC-PDA-ESI-MS, molecular docking, phenolic compounds

## Abstract

**Background/Objectives:** *Dittrichia viscosa* (L.) W. Greuter (Asteraceae) is a Mediterranean medicinal plant whose polar phenolic fraction remains insufficiently characterized. This study aimed to characterize the phenolic composition and evaluate the antioxidant, antimicrobial, and mechanistic bioactivity of aqueous and methanolic leaf extracts collected from the Rmilate Forest in Tangier, northern Morocco. **Methods:** Extracts were characterized by ICP-AES for elemental content and by HPLC-PDA-ESI-MS for phenolic annotation, and total phenolic, flavonoid, and tannin contents were quantified. Antioxidant capacity was assessed using DPPH, ABTS, FRAP, and ORAC assays. Antibacterial activity was evaluated against four reference strains, with time–kill kinetics and membrane integrity assays used to probe the mechanism of action, and antifungal activity was tested against dermatophytic and phytopathogenic fungi. Molecular docking against four bacterial, fungal, and antioxidant enzyme targets and ProTox-3 toxicity prediction were also performed. **Results:** ICP-AES revealed high concentrations of calcium (12,476.75 mg/kg) and potassium (29,699 mg/kg). The methanolic extract showed higher total phenolic (55.05 mg GAE/g), flavonoid (84.18 mg QE/g), and tannin (262.1 mg TAE/g) contents and superior antioxidant activity (DPPH IC_50_: 0.090 mg/mL). Twelve phenolic compounds were tentatively annotated, including caffeic acid, caffeoylquinic acid derivatives, and quercetin/kaempferol glycosides. Antibacterial activity (MIC: 0.25–1 mg/mL) was consistent with cell envelope disruption, and up to 88% mycelial inhibition was achieved against *Trichophyton rubrum*. Docking ranked kaempferol-3-O-pentoside (−10.3 kcal/mol) among the top-scoring ligands, and the top compounds were assigned to toxicity class 5. **Conclusions:** *D. viscosa* is a promising source of bioactive phenolics for pharmaceutical and agrochemical development.

## 1. Introduction

Infectious diseases and oxidative stress-related disorders remain among the most pressing challenges in contemporary biomedical research. The escalating global burden of antimicrobial resistance (AMR), coupled with a critically limited pipeline of novel antibiotics [1,2], has intensified interest in plant-derived bioactive compounds as alternative or complementary therapeutic agents [3]. Simultaneously, the overproduction of reactive oxygen species (ROS) and the resulting oxidative stress have been firmly linked to the pathogenesis of chronic non-communicable diseases, including cardiovascular disorders, type 2 diabetes, neurodegenerative conditions, and cancer [4]. In this context, medicinal plants represent an invaluable reservoir of structurally diverse phytochemicals with demonstrated antioxidant, antimicrobial, and anti-inflammatory properties, offering promising avenues for drug discovery and natural product development.

The Mediterranean basin is recognized as one of the world’s most botanically diverse regions, harboring a rich flora with deep-rooted ethnopharmacological traditions [5]. Among the medicinal plants native to this region, *Dittrichia viscosa* (L.) W. Greuter (syn. *Inula viscosa* (L.) Aiton; Asteraceae) occupies a particularly noteworthy position. This perennial aromatic shrub is widely distributed across the Mediterranean littoral, from the Iberian Peninsula and North Africa to the Middle East, typically colonizing disturbed soils, roadsides, and forest margins. In traditional medicine, *D. viscosa* has been employed for centuries in the treatment of skin infections, wound healing, rheumatic pain, gastrointestinal disorders, and fungal diseases, constituting one of the most ethnobotanically documented species in the Asteraceae family [6].

Previous phytochemical studies of *D. viscosa* have documented a broad spectrum of secondary metabolites—flavonoids, sesquiterpene lactones, diterpenes, caffeic acid derivatives, and essential oils [6,7]—with inuviscolide, tomentosin, quercetin, and luteolin derivatives recognized as key bioactive constituents [8,9].

Among these, tomentosin has been shown to inhibit α-amylase and α-glucosidase, supporting an antidiabetic potential, to exert antifungal activity against dermatophytic fungi including *Microsporum canis* and *Trichophyton mentagrophytes*, and to suppress pro-inflammatory mediators such as IL-6, iNOS, and TNF-α while inhibiting cyclooxygenase-1 and secretory phospholipase A2 [8]. Both tomentosin and inuviscolide have also been reported to induce apoptosis in cancer cell lines through reactive oxygen species generation and activation of intrinsic and extrinsic apoptotic pathways [9]. Quercetin, one of the flavonoid constituents also identified in the present study, is well documented for its potent antioxidant and antibacterial properties, including activity against several of the pathogens investigated here [10,11], while luteolin derivatives have been similarly associated with antioxidant and anti-inflammatory effects [6].

Numerous traditional medicinal plants from Asia, Africa, and South America have been widely studied for their antioxidant and antimicrobial properties, providing valuable reference models for evaluating the therapeutic potential of natural products. Among these, *Camellia sinensis* (green tea, Asia), *Moringa oleifera* (widely distributed across Africa and Asia), and *Uncaria tomentosa* (cat’s claw, South America) are particularly recognized for their rich polyphenolic composition and broad spectrum of biological activities.

However, most work has focused on essential oil fractions or limited analytical approaches, leaving the polar phenolic fraction relatively undercharacterized. Likewise, while antimicrobial activity has been reported, systematic evaluations integrating time–kill kinetics, minimum bactericidal concentration determination, membrane integrity assessment, and antifungal dose–response profiling remain scarce, limiting mechanistic understanding of the mode of action.

Northern Morocco, and the Tangier–Tetouan–Al Hoceima region in particular, sits at the meeting point of Mediterranean and Atlantic climatic zones, a position that has long been linked to notable phytochemical variability among native plant populations [12]. Given this ecological distinctiveness, populations of widespread species such as *D. viscosa* growing in this area may harbor metabolite profiles that differ meaningfully from those documented elsewhere in the Mediterranean [13]. Yet despite the plant’s well-established place in the traditional medicine of the region, few integrated studies have combined elemental analysis by ICP-AES, phenolic profiling by HPLC-PDA-ESI-MS, mechanistic antimicrobial evaluation including time–kill kinetics and membrane integrity assays, antifungal dose–response profiling, molecular docking against validated enzyme targets, and in silico toxicity prediction on material collected in northern Morocco.

The present study was therefore designed to address this gap comprehensively by characterizing the elemental profile of *D. viscosa* leaves by ICP-AES, quantifying total phenolic, flavonoid, and tannin contents and identifying individual phenolic compounds by HPLC-PDA-ESI-MS, evaluating antioxidant capacity using four complementary assays (DPPH, ABTS, FRAP, and ORAC), and assessing antibacterial activity through agar well diffusion, broth microdilution, time–kill kinetics, and membrane integrity assays. Antifungal activity was further evaluated against dermatophytic and phytopathogenic fungal strains, while molecular docking of the tentatively annotated phenolic constituents against four validated bacterial and fungal enzyme targets and in silico toxicity profiling of the top-ranked hits using ProTox-3 were performed to extend the experimental findings to the molecular scale. Together, these investigations establish a composition–activity framework for *D. viscosa* polyphenols and provide a mechanistically grounded basis for their further development as natural bioactive agents.

Although previous studies have separately reported the phytochemical composition or biological activities of *Dittrichia viscosa*, to the best of our knowledge, the present work provides an integrated evaluation combining comprehensive phenolic profiling by HPLC-PDA-ESI-MS with mechanistic antibacterial investigations, antifungal evaluation, antioxidant characterization, molecular docking, and in silico toxicity prediction using leaf material collected from northern Morocco. Rather than focusing on a single biological endpoint, this study establishes a composition–activity framework that highlights the phenolic constituents most likely to contribute to the observed bioactivities and provides a rational basis for future bioassay-guided isolation and experimental validation.

## 2. Results and Discussion

The present study combined elemental characterization, phytochemical profiling, antioxidant evaluation, mechanistic antimicrobial assays, antifungal testing, and molecular docking to establish relationships between the chemical composition of *D. viscosa* and its biological activities. Rather than evaluating each dataset independently, the objective was to integrate these complementary approaches to obtain a more comprehensive understanding of the contribution of the phenolic fraction to the observed bioactivities.

### 2.1. Trace Elemental Analysis by ICP-AES

The information in Table 1 pertains to the elemental characterization of the *Dittrichia viscosa* leaves. Analysis of *Dittrichia viscosa* leaves using ICP-AES revealed the presence of substantial concentrations of crucial elements, including calcium (Ca), magnesium (Mg), iron (Fe), and potassium (K), which are vital for various physiological processes such as cell structure formation, chlorophyll production, and enzyme activation [14,15]. Manganese (Mn), copper (Cu), and zinc (Zn) are trace elements and were also detected; they contribute to metabolic pathways and cellular functions [15,16].

Chromium (Cr) and nickel (Ni) are reported as not detected (−) in Table 1, as their concentrations fell below the instrument detection limits under the analytical conditions employed. The purpose of this analysis was to provide a comprehensive elemental profile of the investigated Moroccan *D. viscosa* population, complementing its phytochemical characterization and contributing to the quality assessment of this medicinal plant. Although elemental composition may vary according to geographical origin, environmental conditions, or plant developmental stage, the present study focused on the characterization of a single well-defined population. Comparative investigations involving different growth stages or collection periods represent an interesting direction for future research. Overall, these findings enhance our understanding of the nutritional value and potential applications of *Dittrichia viscosa* in agriculture, nutrition, and herbal medicine, highlighting its significance in various fields.

### 2.2. Phytochemical Screening

**Figure 1:**
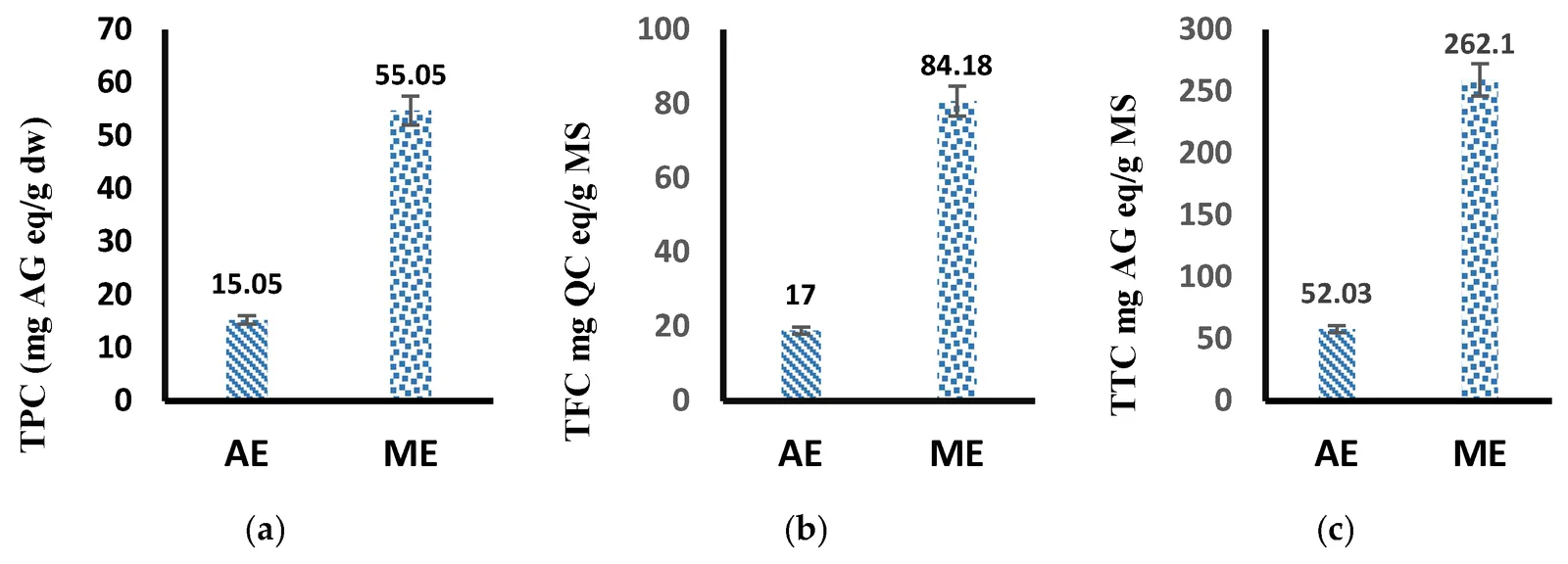
Quantitative phytochemical analysis of *Dittrichia viscosa* extracts (**a**) TPC, (**b**) TFC, and (**c**) TTC. **AE:** aqueous extract; **ME:** methanolic extract.

Figure 1 displays the phenolic compounds, flavonoids, and tannins present in *Dittrichia viscosa* leaf extracts. Our findings revealed a significantly higher total phenol content (TPC), total flavonoid content (TFC), and total tannin content (TTC) in the methanolic extract than in the aqueous extract. In particular, the methanolic extract exhibited a significantly higher TPC (55.05 mg GAE/g extract) than the aqueous extract’s lower TPC of 15.05 mg GAE/g extract. Similarly, the TFC in the methanolic extract was substantially higher at 84.18 mg QE/g extract, as compared with the aqueous extract’s 17 mg QE/g extract. Furthermore, TTC was markedly elevated in the methanolic extract (262.1 mg TAE/g extract compared with that of the aqueous extract’s 52.03 mg TAE/g extract). These results suggest that methanolic extraction solvents are more effective than aqueous solvents for extracting phenolic compounds, flavonoids, and tannins from *Dittrichia viscosa*. The use of alcohol-water mixtures has been proven to be more effective for extracting phenolic compounds than single-component solvent systems. By combining an organic solvent with a small amount of water, a more polar environment is created, which enhances the polyphenol extraction [17,18]. Therefore, the high concentration of phenolic compounds observed in the 80% (*v*/*v*) methanol–water extract can be attributed to the increased polarity of the solvent, which boosts the extraction efficiency.

### 2.3. Functional Groups Identification in Methanolic Extract by FTIR Analysis

FTIR spectroscopy is a widely used technique that is commonly applied in various fields, including food analysis, environmental monitoring, pharmaceuticals, and biomedical research [18]. It is recognized for its ability to rapidly characterize functional groups and provide complementary structural information on complex plant matrices. Although FTIR cannot identify individual metabolites in crude extracts, it serves as a valuable preliminary tool for characterizing the major functional groups present and complements chromatographic and mass spectrometric analyses [19]. FTIR analysis can aid in understanding the therapeutic potential of these metabolites and provides a non-destructive means of studying plant biomass and its biochemical composition.

**Figure 2:**
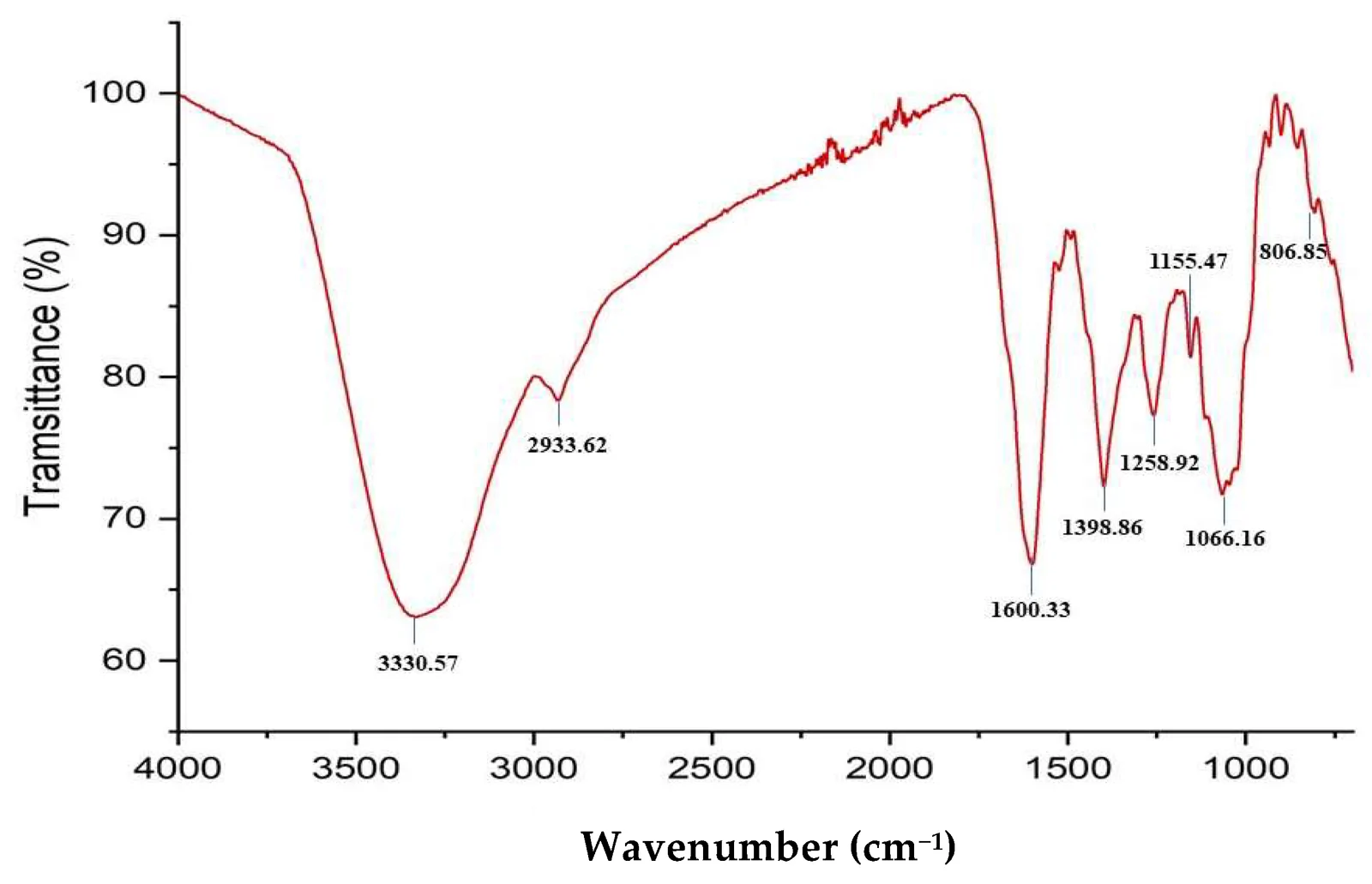
IR spectra of methanolic extract.

The infrared spectrum of the methanolic extract of *Dittrichia viscosa* presented several distinct peaks, revealing the presence of specific functional groups within the sample (Figure 2). The infrared spectrum of the methanolic extract of *Dittrichia viscosa* exhibited characteristic absorption bands corresponding to hydroxyl, aliphatic, aromatic, carbonyl, and C–O-containing functional groups, reflecting the complex chemical composition of the crude extract. The prominent peak detected at 3330.57 cm^−1^ correlates with the stretching vibration of O–H bonds, suggesting the presence of hydroxyl groups, commonly found in alcohols or phenols. Additionally, the peak at 2933.62 cm^−1^ indicates the stretching vibration of C–H bonds in aliphatic compounds such as alkyl groups. The absorption band observed at 1600.33 cm^−1^ is mainly attributed to aromatic C=C stretching vibrations; however, in polyphenol-rich plant extracts, it may also include contributions from conjugated carbonyl (C=O) stretching of phenolic acids and flavonoids. The position and intensity of these bands can be influenced by hydrogen bonding and overlapping absorptions arising from the complex composition of the extract. This interpretation is consistent with the HPLC-PDA-ESI-MS analysis, which revealed caffeic acid derivatives, caffeoylquinic acids, quercetin glycosides, and kaempferol glycosides, all containing conjugated carbonyl functionalities. The bands at 1258.92, 1155.47, and 1066.16 cm^−1^ are more appropriately assigned to C–O stretching vibrations of phenolic compounds, ethers, esters, and glycosidic linkages rather than to carbonyl stretching. Finally, the peak at 806.85 cm^−1^ could indicate additional C–H bending vibrations in the aromatic compounds. These interpretations collectively provide valuable insights into the chemical composition and structural characteristics of *Dittrichia viscosa* methanolic leaf extract.

### 2.4. Antioxidant Activity

**Figure 3:**
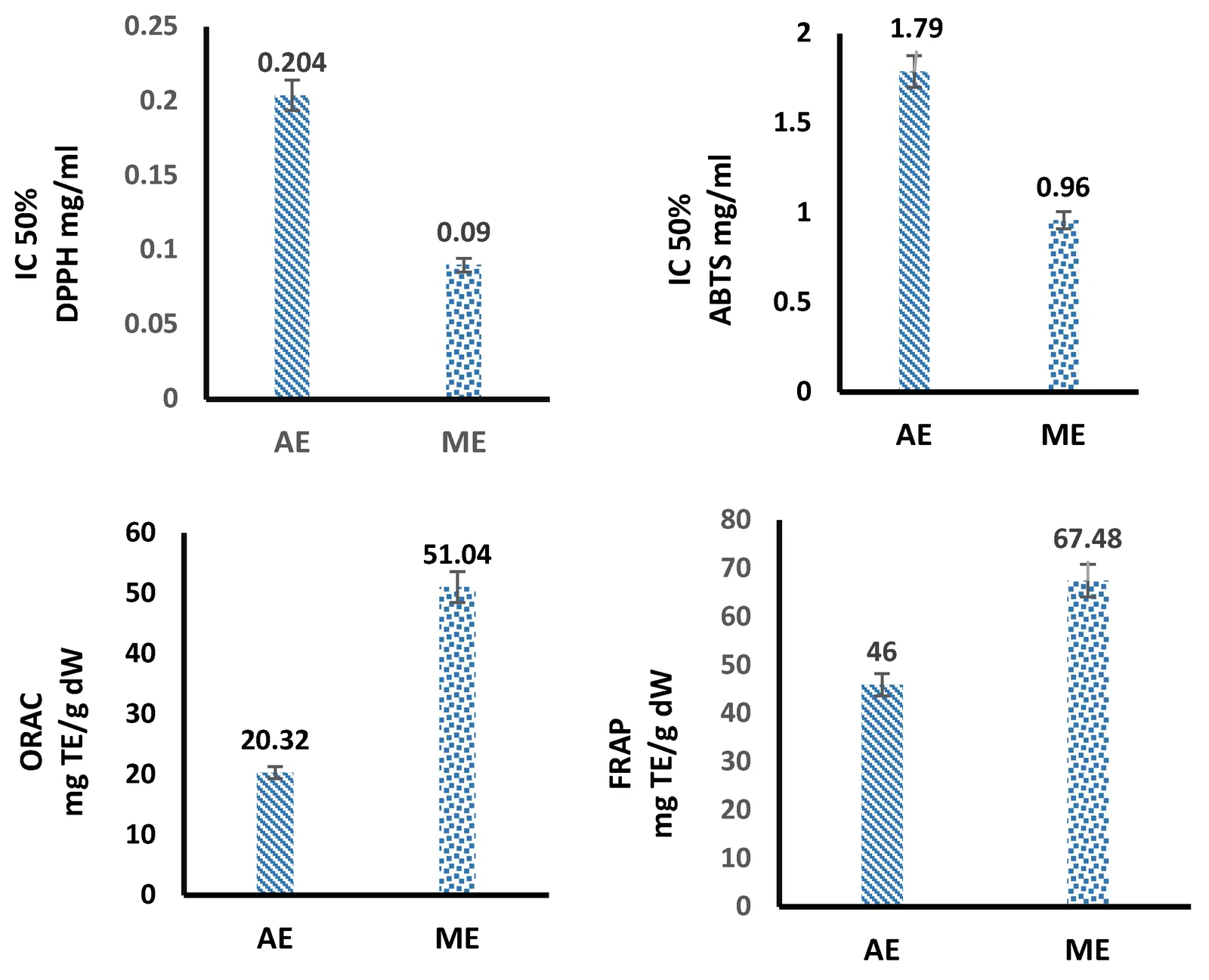
Antioxidant activity of *D. viscosa* extracts using DPPH, ABTS, FRAP and ORAC assays. **AE:** aqueous extract; **ME:** methanolic extract.

To this end, we assessed the antioxidant capacity of *Dittrichia viscosa* leaf extracts using different methods, such as their ability to bind the free radical DPPH, cation ABTS, and Oxygen Radical Absorbance Capacity (ORAC), and their antioxidative ferric reductase activity (FRAP). Figure 3 presents the results.

Comparison of the antioxidant properties of the methanolic and aqueous extracts of *Dittrichia viscosa* revealed noticeable variations across multiple assays. In the DPPH assay, which measures the ability of antioxidants to neutralize free radicals, the methanolic extract demonstrated significantly higher activity, with an IC50 value of 0.090 ± 0.007 mg/mL, compared with the aqueous extract IC50 value of 0.204 ± 0.005 mg/mL. This suggests that the methanolic extract exhibited stronger free radical scavenging potential. Similarly, in the ABTS assay, which also measures radical scavenging activity, the methanolic extract outperformed the aqueous extract with an IC50 value of 0.96 ± 0.02 mg/mL, whereas the aqueous extract showed a higher IC50 value of 1.79 ± 0.07 mg/mL. Moreover, the methanolic extract demonstrated superior antioxidant capacity in the FRAP assay (67.48 ± 0.1 mg TE/g dW) compared with the aqueous extract (46 ± 0.1 mg TE/g dW), indicating its ability to reduce ferric ions. Similarly, in the ORAC assay, a technique that assesses antioxidant capacity against peroxyl radicals, the methanolic extract displayed greater activity (51.04 ± 0.7 mg TE/g dW) compared with the aqueous extract (20.32 ± 0.7 mg TE/g dW). These results indicate that the methanolic extract of *Dittrichia viscosa* possesses stronger antioxidant properties than its aqueous counterpart, suggesting its potential as a valuable source of natural antioxidants.

The stronger antioxidant activity exhibited by the methanolic extract is consistent with its higher phenolic, flavonoid, and tannin contents (Figure 1). HPLC-PDA-ESI-MS analysis further revealed the presence of several well-known antioxidant phenolics, including caffeic acid derivatives, dicaffeoylquinic acids, quercetin glycosides, and kaempferol glycosides. These compounds possess multiple hydroxyl groups capable of donating hydrogen atoms or electrons to neutralize free radicals and reduce oxidizing species. Therefore, the antioxidant activity observed in the methanolic extract is likely attributable to the combined contribution of these phenolic constituents rather than to a single compound.

### 2.5. Phenolic Profile of Dittrichia viscosa Methanolic Extract by HPLC-PDA/ESI-MS Analysis

**Figure 4:**
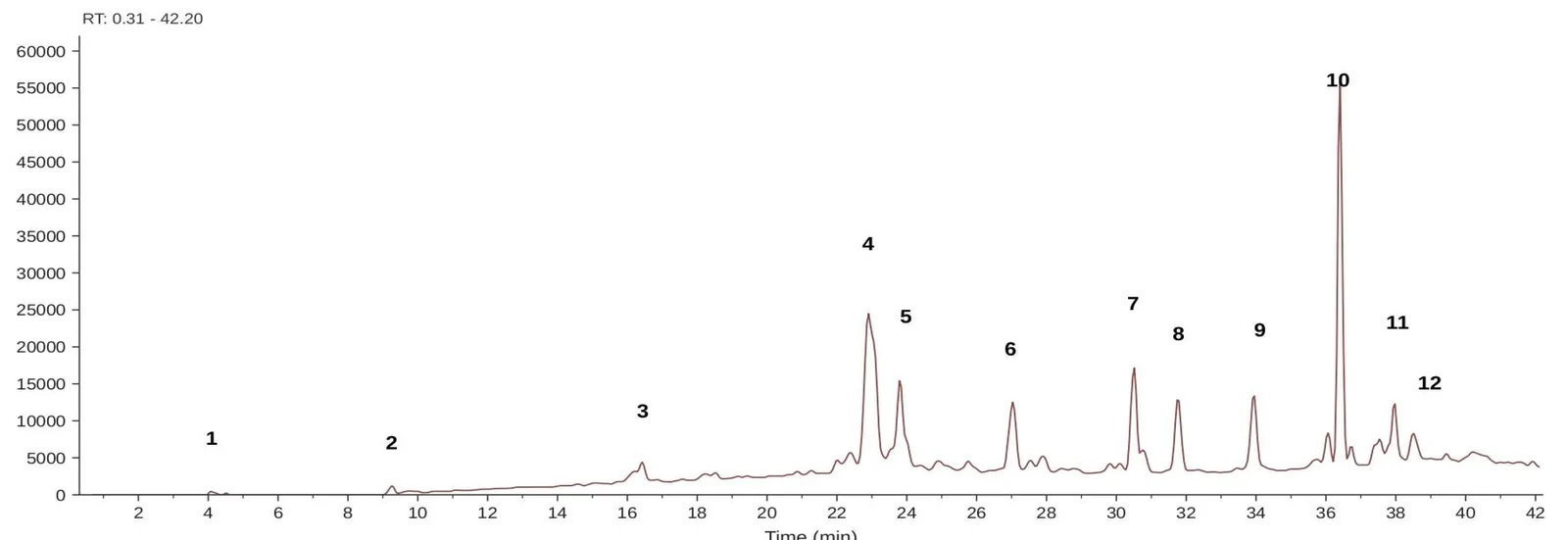
Chromatographic profile of *Dittrichia viscosa* leaves methanolic extract by HPLC.

Two ionization techniques, positive and negative, were employed to perform HPLC-ESI-MS analyses in order to gain a deeper understanding of the composition of *Dittrichia viscosa* methanolic extract. The negative ion mode yielded the most favorable results and is discussed below. The chromatographic profile of the methanolic extract of *Dittrichia viscosa* is depicted in Figure 4, while Table 2 provides putative identification based on retention time, maximum absorption wavelength and precursor ions.

HPLC analysis of the methanolic extract from *Dittrichia viscosa* leaves revealed a diverse array of phytochemical constituents, resulting in the identification of 12 compounds. Among these, caffeic acid was detected at 3.98 min, exhibiting characteristic absorption at 273 nm and an [M-H]^−^ ion at *m*/*z* 179. Shikimic acid, eluting at 9.03 min, displayed an absorption maximum at 254 nm and an [M-H]^−^ ion at *m*/*z* 173. Caffeoylquinic acid, observed at 16.31 min, exhibited absorption peaks at 320 and 330 nm, along with an [M-H]^−^ ion at *m*/*z* 353. Dihydroxybenzoic acid, found at 23.73 min, showed absorption at 240 nm and an [M-H]^−^ ion at *m*/*z* 153. Quercetin-O-glucuronide, detected at 23.92 min, demonstrated absorption at 370 nm along with an [M-H]^−^ ion at *m*/*z* 477, confirming its flavonoid nature. Quercetin-O-hexoside 1 and Quercetin-O-hexoside 2, eluting at 27.87 and 30.73 min, respectively, exhibited absorption at 370 nm and [M-H]^−^ ions at *m*/*z* 463, representing the glycosides of quercetin. Kaempferol-7-O-hexoside, at 31.73 min, exhibited absorption at 365 nm and an [M-H]^−^ ion at *m*/*z* 447, which indicated its identity as a glycosylated form of kaempferol. Similarly, kaempferol-3-O-pentoside, eluted at 33.91 min, showed absorption at 365 nm and an [M-H]^−^ ion at *m*/*z* 417, confirming its nature as another flavonoid glycoside. Moreover, di-O-caffeoylquinic acid, iso-di-O-caffeoylquinic acid I, and iso-di-O-caffeoylquinic acid II, detected at retention times of 36.71, 37.96, and 38.50 min, respectively, exhibited absorption peaks around 325–330 nm along with [M-H]^−^ ions at *m*/*z* 515, which indicated their identity as isomeric caffeoylquinic acids. Collectively, these findings offer valuable insights into the diverse phytochemical profile of *Dittrichia viscosa* leaves, which may have potential therapeutic applications because of their bioactive constituents.

The importance of identifying these 12 compounds in the methanolic extract of *Dittrichia viscosa* leaves lies in their diverse range of medicinal and antioxidant activities. Caffeic acid is well known for its potent antioxidant and anti-inflammatory properties, which are crucial for combating oxidative stress and inflammation-related disorders [20,21]. Shikimic acid, an intermediate in the biosynthesis of aromatic amino acids, is of great interest because of its potential role in plant defence mechanisms and pharmaceutical applications in the production of antiviral drugs [22,23]. Caffeoylquinic acid derivatives have diverse biological activities, including antioxidant, anti-inflammatory, and hepatoprotective effects [24]. In addition, they possess the ability to modulate glucose-stimulated insulin secretion (GSIS) and α-glucosidase activity, contributing significantly to the medicinal value of the extract [25]. Flavonoids such as quercetin and kaempferol glycosides are known for their antioxidant, anti-cancer, anti-inflammatory, and neuroprotective properties, making them promising candidates for therapeutic interventions against various diseases [26,27]. Overall, the identification of these compounds highlights the pharmacological potential of *Dittrichia viscosa* leaves, supporting further investigation of their therapeutic applications in medicine and natural product-based drug development.

The HPLC-PDA-ESI-MS method employed in this study was specifically designed for the characterization of phenolic constituents. Although twelve major phenolic constituents were tentatively annotated, we cannot exclude the contribution of unidentified metabolites to the observed biological activities. The antioxidant and antimicrobial properties of crude plant extracts frequently arise from additive or synergistic interactions among multiple constituents rather than from a single compound. Consequently, the compounds identified in the present work should be regarded as the principal candidates contributing to the observed bioactivities. Based on the combination of literature-reported biological activities and the molecular docking results obtained in the present study, kaempferol-3-O-pentoside, kaempferol-7-O-hexoside, and di-O-caffeoylquinic acid emerged as the most promising candidate bioactive constituents and were therefore selected for further in silico evaluation. Nevertheless, future bioassay-guided fractionation, purification, and experimental validation will be necessary to confirm their individual contributions to the biological activities of the crude extract.

### 2.6. Antimicrobial Activity

#### 2.6.1. Antibacterial Activity

The antibacterial activity of *Dittrichia viscosa* leaf extracts was assessed using the agar well diffusion method and broth microdilution, and the results are presented in Table 3 and Table 4. Both extracts demonstrated antibacterial activity against all four tested strains, with the methanolic extract consistently outperforming the aqueous extract across all parameters.

In the agar well diffusion assay (results summarized in Table 3; gentamicin 10 µg/mL positive control inhibition zones ranged from 18.0 to 28.5 mm across the four strains), the methanolic extract produced the largest inhibition zones against *B. subtilis* (20.4 ± 0.9 mm) and *S. aureus* (18.2 ± 0.8 mm), followed by *E. coli* (16.8 ± 0.8 mm) and *P. aeruginosa* (13.5 ± 0.7 mm). The aqueous extract yielded significantly smaller inhibition zones across all strains (*p* < 0.05), ranging from 8.4 ± 0.5 mm (*P. aeruginosa*) to 13.1 ± 0.7 mm (*B. subtilis*).

MIC values for the methanolic extract ranged from 0.25 mg/mL (*B. subtilis*) to 1 mg/mL (*P. aeruginosa*), while aqueous extract MIC values were 4- to 8-fold higher, ranging from 1 mg/mL (*B. subtilis*) to 4 mg/mL (*P. aeruginosa*). MBC values were consistently twice the corresponding MIC for both extracts across all strains, yielding MBC/MIC ratios of 2 throughout. The lowest MIC value was recorded for the methanolic extract against *B. subtilis* (0.25 mg/mL), indicating strong bactericidal potency against this organism.

The antibacterial results obtained by both agar well diffusion and broth microdilution methods consistently demonstrate that the methanolic extract of *Dittrichia viscosa* possesses significantly stronger antibacterial activity than the aqueous extract, a finding that aligns with its markedly higher phenolic, flavonoid, and tannin content established in the phytochemical screening (Figure 2). The greater extractive efficiency of methanol–water mixtures for polyphenolic compounds is well documented and directly underpins this differential activity [28,29].

The pattern of susceptibility observed across bacterial strains—with Gram-positive organisms (*B. subtilis* and *S. aureus*) consistently more sensitive than Gram-negative species (*E. coli* and *P. aeruginosa*)—is in accordance with well-established differences in bacterial cell wall architecture. The additional outer membrane of Gram-negative bacteria, rich in lipopolysaccharides, constitutes a formidable permeability barrier that limits the intracellular accumulation of polyphenolic compounds [30]. The notably high MIC of the methanolic extract against *P. aeruginosa* (1 mg/mL) compared with *B. subtilis* (0.25 mg/mL) reflects this organism’s exceptional intrinsic resistance, which is further reinforced by active efflux pump systems and reduced outer membrane permeability [31].

The uniform MBC/MIC ratio of 2 across all strains and both extracts is a particularly meaningful finding. A MBC/MIC ratio ≤ 4 is the accepted threshold for classifying an antimicrobial agent as bactericidal rather than bacteriostatic. The consistent ratio of 2 observed here therefore confirms that both extracts exert a bactericidal mode of action against all tested organisms, not merely inhibiting growth but actively killing bacterial cells. This is clinically significant, as bactericidal agents are generally preferred over bacteriostatic ones in the treatment of severe or immunocompromised infections [32]. Furthermore, this uniform ratio across structurally distinct Gram-positive and Gram-negative organisms suggests that the extract acts through a non-specific membrane-disrupting mechanism rather than a single enzymatic target—a hypothesis directly supported by the nucleic acid and protein leakage data reported in the present study, where significant release of intracellular contents was observed following treatment at 1× MIC.

#### 2.6.2. Time–Kill Kinetics

**Figure 5:**
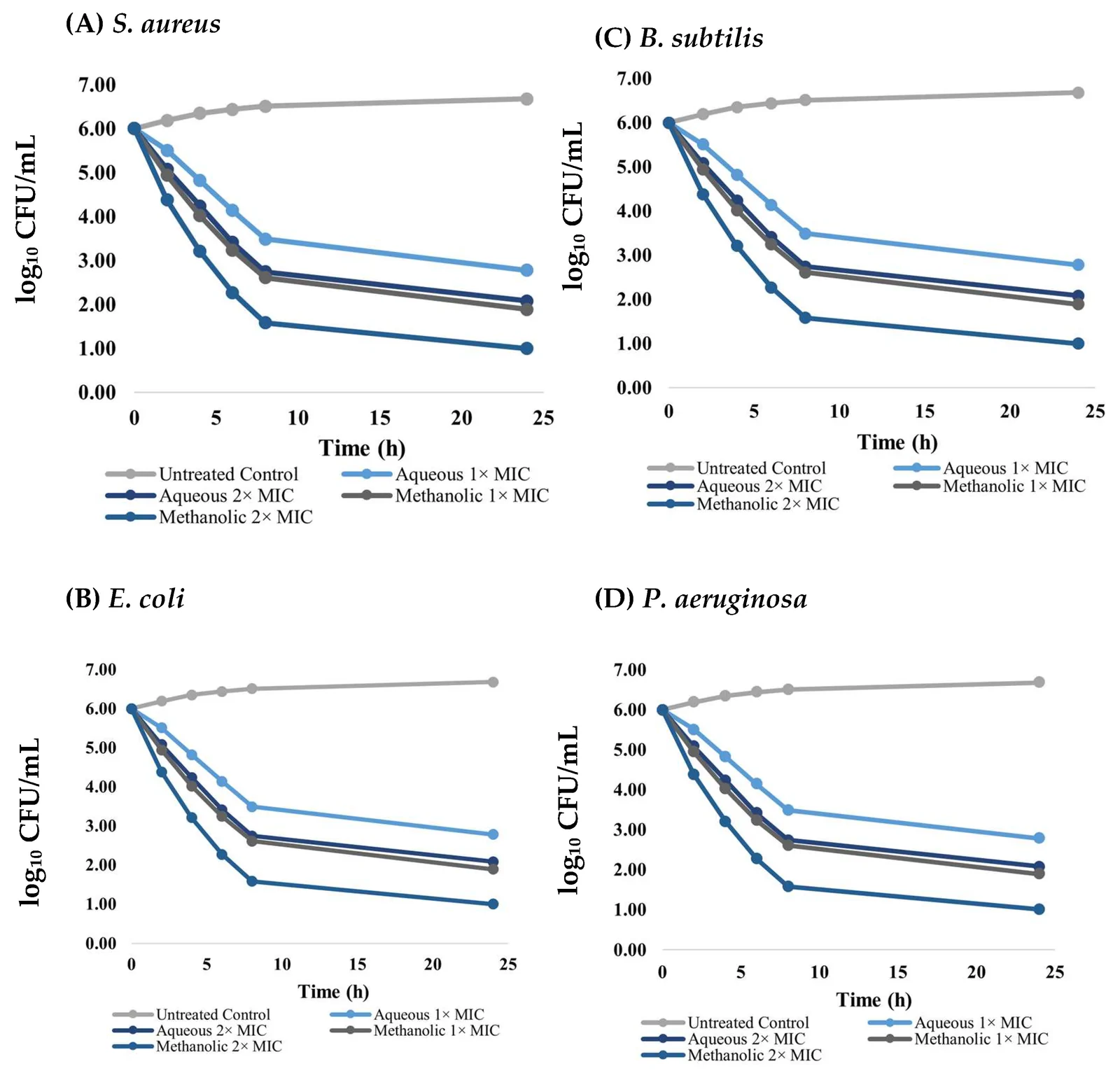
Time–kill kinetics of *D. viscosa* aqueous and methanolic extracts against four bacterial strains. (**A**) *S. aureus*, (**B**) *E. coli*, (**C**) *B. subtilis*, (**D**) *P. Aeruginosa*.

Time–kill analysis was conducted to characterize the bactericidal dynamics of *Dittrichia viscosa* extracts beyond the information provided by static MIC determinations, allowing assessment of both the rate and magnitude of bacterial killing as a function of time and concentration. All four strains exhibited progressive, concentration-dependent reductions in viable counts, confirming that the antibacterial activity of both extracts follows a time- and concentration-dependent killing pattern (Figure 5).

Against the Gram-positive strains, the methanolic extract at 2× MIC reduced viable counts of *S. aureus* and *B. subtilis* from 6.00 log_10_ CFU/mL to 1.00 and 1.00 log_10_ CFU/mL, respectively by 24 h, representing a 5.00 log_10_ reduction that substantially exceeded the 3 log_10_ bactericidal threshold. Notably, bactericidal activity was already approached at 8 h (reductions of 4.42 and 4.42 log_10_ for *S. aureus* and *B. subtilis*, respectively), indicating rapid and sustained killing kinetics. The aqueous extract at 2× MIC also achieved bactericidal activity at 24 h against both Gram-positive strains, with reductions of 3.92 and 3.92 log_10_ CFU/mL, though the killing rate was consistently slower than that of the methanolic extract at equivalent concentrations.

The Gram-negative strains demonstrated markedly attenuated susceptibility throughout the observation period. Against *E. coli*, the methanolic extract at 2× MIC produced a reduction of 4.53 log_10_ CFU/mL at 24 h, achieving bactericidal activity, while *P. aeruginosa* reached a reduction of only 4.32 log_10_ CFU/mL under the same conditions. The aqueous extract at 2× MIC failed to surpass the bactericidal threshold against *P. aeruginosa* at any timepoint, with a maximum reduction of 3.42 log_10_ CFU/mL at 24 h. This differential response between Gram-positive and Gram-negative organisms is mechanistically consistent with the structural complexity of the Gram-negative cell envelope, whose outer membrane—composed of lipopolysaccharide and porin proteins—restricts passive diffusion of polyphenolic compounds and limits their access to the cytoplasmic membrane and intracellular targets [33]. *P. aeruginosa* in particular is known to exhibit intrinsic multidrug resistance through constitutive expression of efflux pumps and reduced outer membrane permeability [34], which likely contributed to its relatively diminished response even at 2× MIC.

At 1× MIC, neither extract achieved bactericidal activity against any strain within the 24 h observation period, with viable count reductions consistently falling below the 3 log_10_ threshold. This concentration-dependent transition from bacteriostatic to bactericidal behavior—corroborated by the uniform MBC/MIC ratio of 2 across all strains—is characteristic of membrane-active agents whose lethal effects require a critical accumulation at the cell surface. At sublethal concentrations, polyphenols disrupt membrane potential, inhibit ATPase activity, and interfere with nutrient transport without inducing irreversible structural damage [35], whereas at bactericidal concentrations the cumulative membrane perturbation exceeds the cell’s capacity for repair and recovery.

The untreated control maintained stable viable counts across all timepoints (ranging from 6.00 to 6.78 log_10_ CFU/mL at 24 h across strains), confirming the absence of nutritional depletion, spontaneous die-off, or environmental stress artifacts, and validating the experimental conditions throughout the assay.

#### 2.6.3. Membrane Integrity Assay

**Figure 6:**
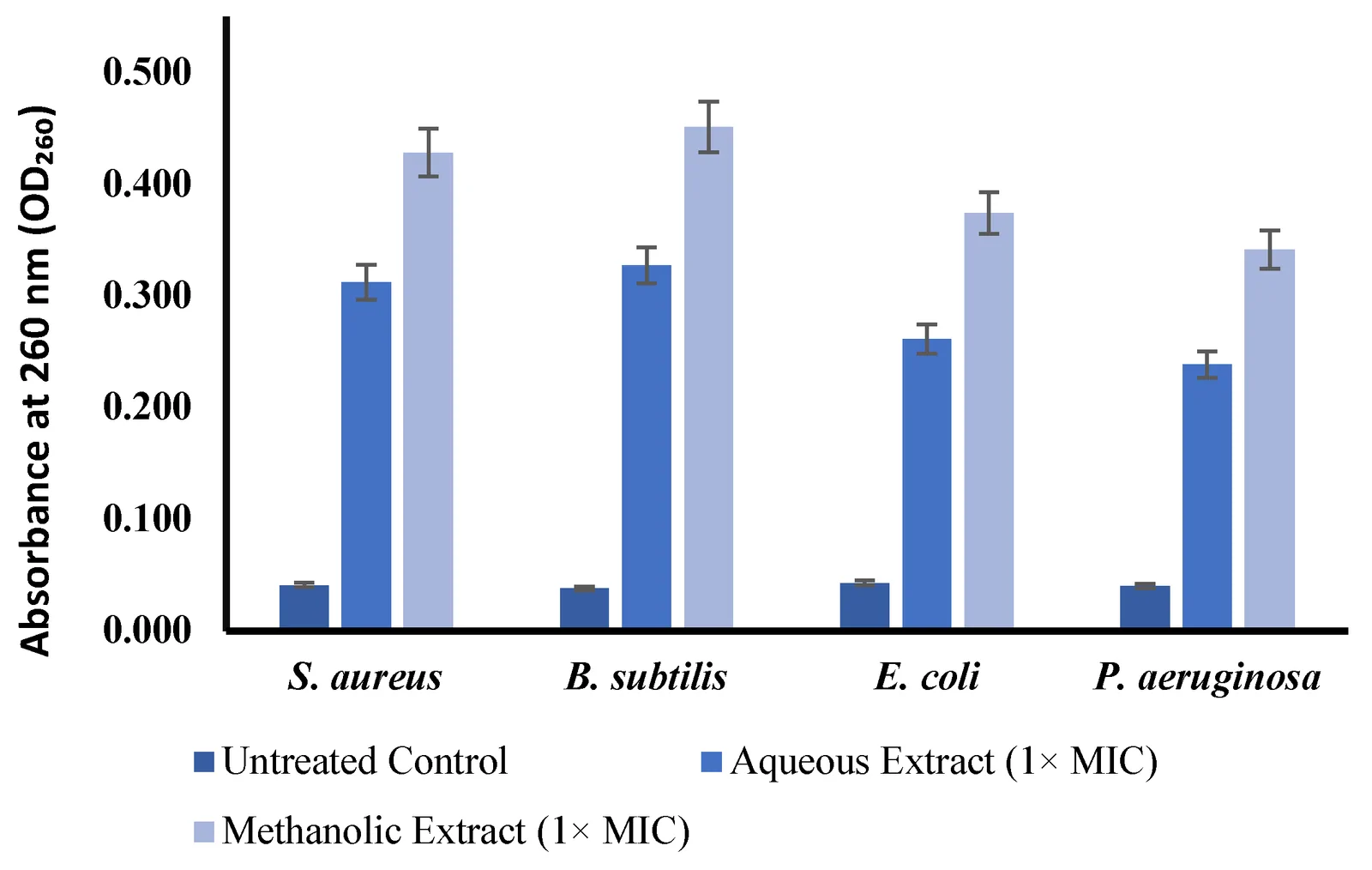
Membrane integrity of bacterial strains treated with *D. viscosa* extracts at 1× MIC, measured by OD_260_.

Measurement of intracellular macromolecule (nucleic acid and protein) release at 260 nm revealed a pronounced and statistically significant elevation in OD_260_ across all four bacterial strains following treatment with both extracts at 1× MIC for 4 h, relative to untreated controls (*p* < 0.05) (Figure 6). These results strongly suggest bacterial cell membrane disruption as a primary lethal mechanism, although the OD_260_-based assay measures total nucleic acid and protein leakage and cannot distinguish individual intracellular components; complementary techniques (e.g., propidium iodide staining or electrolyte release measurement) would further corroborate this interpretation.

Gram-positive strains sustained the greatest degree of membrane damage. Against *S. aureus* and *B. subtilis*, the methanolic extract yielded OD_260_ values of 0.428 ± 0.012 and 0.451 ± 0.015, corresponding to 10.4- and 11.9-fold increases over untreated controls (0.041 ± 0.003 and 0.038 ± 0.004, respectively). The aqueous extract produced comparably significant but quantitatively lower leakage (0.312 ± 0.009 and 0.327 ± 0.011 for *S. aureus* and *B. subtilis*, respectively), a difference directly attributable to its substantially reduced polyphenolic content relative to the methanolic extract (Figure 2). Against *E. coli* and *P. aeruginosa*, leakage remained statistically significant yet of considerably lower magnitude: OD_260_ values of 0.374 ± 0.013 and 0.341 ± 0.010 were recorded for the methanolic extract, and 0.261 ± 0.009 and 0.238 ± 0.008 for the aqueous extract, against baseline control readings of 0.043 ± 0.003 and 0.040 ± 0.002.

The attenuated leakage in Gram-negative organisms reflects the well-established barrier function of the outer LPS-phospholipid membrane, which restricts passive diffusion of polyphenolic compounds toward the cytoplasmic membrane [33]. *P. aeruginosa* is an extreme case: constitutive MexAB-OprM and MexXY-OprM efflux systems combined with reduced OprD porin expression substantially curtail intracellular polyphenol accumulation [36]. That measurable leakage still occurred at 1× MIC in this organism (OD_260_: 0.341 ± 0.010 vs. control 0.040 ± 0.002) confirms genuine membrane-disrupting potency even against one of the most intrinsically resistant clinical pathogens.

The compounds most likely responsible are the dicaffeoylquinic acid isomers (peaks 10–12), caffeic acid (peak 1), and quercetin and kaempferol glycosides (peaks 5–9). Caffeic acid and quinic acid conjugates are amphiphilic: at bactericidal concentrations they intercalate into the phospholipid bilayer, disrupt lateral lipid packing, and dissipate the proton motive force, collapsing the electrochemical gradient essential for ATP synthesis and ion homeostasis [37,38]. Quercetin and kaempferol glycosides act complementarily by inhibiting membrane-bound ATPases [39,40] and chelating divalent cations (Mg^2+^, Ca^2+^) that stabilise LPS, thereby potentiating caffeic acid penetration through a self-promoted uptake pathway [10,41]. This multi-target synergy explains the broad bactericidal activity across organisms as divergent as *B. subtilis* and *P. aeruginosa* [42].

### 2.7. Antifungal Activity

The antifungal activity of *Dittrichia viscosa* leaf extracts was evaluated against two dermatophytic fungi (*Trichophyton rubrum* and *Microsporum canis*) and two phytopathogenic fungi (*Botrytis cinerea* and *Fusarium oxysporum*) using the direct contact method, and results are expressed as percentage inhibition of mycelial growth (Table 5). Both extracts exhibited dose-dependent antifungal activity against all tested strains, with inhibition increasing progressively with concentration from 0.5 to 4.0 mg/mL (*p* < 0.05).

The methanolic extract demonstrated significantly stronger antifungal activity than the aqueous extract at all tested concentrations (*p* < 0.05). At the highest concentration tested (4.0 mg/mL), the methanolic extract achieved maximum mycelial inhibition against *T. rubrum* (88.0 ± 3.0%), followed by *M. canis* (84.0 ± 3.0%), *B. cinerea* (68.0 ± 4.0%), and *F. oxysporum* (62.0 ± 4.0%). Even at the lowest concentration (0.5 mg/mL), the methanolic extract produced notable inhibition ranging from 18.0 ± 2.0% (*F. oxysporum*) to 35.0 ± 3.0% (*T. rubrum*), indicating meaningful antifungal potency at sub-maximal doses. The aqueous extract similarly displayed dose-dependent activity, though at considerably lower levels, with inhibition at 4.0 mg/mL ranging from 38.0 ± 3.0% (*F. oxysporum*) to 63.0 ± 4.0% (*T. rubrum*). Across both extracts and all concentrations, dermatophytic fungi (*T. rubrum* and *M. canis*) were consistently more susceptible than phytopathogenic species (*B. cinerea* and *F. oxysporum*), and *F. oxysporum* exhibited the lowest susceptibility in all conditions.

The superior antifungal activity of the methanolic extract is directly attributable to its higher phenolic content (Figure 2). Polyphenols exert antifungal effects through disruption of membrane integrity via interaction with ergosterol, inhibition of cell wall biosynthesis, interference with mitochondrial function, and suppression of spore germination [43]. Quercetin inhibits fungal topoisomerase II and disrupts membrane permeability in dermatophytes [37,44], while caffeoylquinic acids inhibit glucan synthase [45], collectively accounting for the dose-dependent mycelial inhibition recorded here.

The greater susceptibility of dermatophytes (*T. rubrum* and *M. canis*) relative to phytopathogenic species reflects their limited metabolic flexibility and narrower cell wall composition [43]. *F. oxysporum* showed the lowest susceptibility, consistent with its robust detoxification machinery—including laccase and peroxidase systems that degrade phenolic compounds—rather than an absence of antifungal potency [11]. The antifungal values reported here are broadly consistent with published data on *D. viscosa* essential oils [46] and methanolic extracts [47,48], supporting the differential susceptibility pattern observed.

The antifungal values reported here compare favorably with data from related Asteraceae species. Ref. [46] reported antimicrobial activity for *Dittrichia viscosa* essential oil against fungal pathogens at comparable concentrations, while [48] recorded significant inhibition against dermatophytic species for methanolic extracts of *D. viscosa* (syn. *Inula viscosa*), broadly consistent with the present findings. Ref. [47] similarly demonstrated that the methanolic extract of *D. viscosa* exhibited the highest antifungal activity among tested extracts, with particular efficacy against *M. canis*, further supporting the differential susceptibility pattern observed here. These results collectively highlight the potential of *D. viscosa* methanolic leaf extract as a dual-action antimicrobial agent with both antibacterial and antifungal applications, warranting further investigation into its development as a natural preservative or therapeutic agent [42,43].

Although the methanolic extract contained considerably higher levels of total phenolics, flavonoids, and tannins than the aqueous extract, antifungal activity is not necessarily proportional to the total concentration of these phytochemicals. Biological activity depends on the qualitative composition of the extract, the intrinsic activity of individual compounds, and potential synergistic or antagonistic interactions among phenolic and non-phenolic constituents. Therefore, the moderate antifungal activity observed for the aqueous extract may reflect the presence of specific bioactive metabolites rather than the overall abundance of phenolic compounds.

### 2.8. Molecular Docking

Molecular docking analysis was performed as a complementary computational approach to prioritize the major phenolic constituents identified by HPLC-PDA-ESI-MS according to their predicted interactions with biologically relevant targets. Because the biological activities of crude plant extracts generally result from synergistic interactions among multiple constituents, the objective of this analysis was not to identify a single definitive active compound but rather to determine which phenolic constituents are the most likely contributors to the experimentally observed antioxidant, antibacterial, and antifungal activities. Accordingly, molecular docking was used as a hypothesis-generating and compound-prioritization tool, providing mechanistic support for the experimental findings while identifying the most promising candidate bioactive compounds for future bioassay-guided isolation and pharmacological validation. The target panel comprised four validated enzymes: *S. aureus* tyrosyl-tRNA synthetase (1JIL) and *E. coli* DNA gyrase B (5L3J) as mechanistically complementary antibacterial targets; *C. albicans* sterol 14α-demethylase (5TZ1) as a canonical antifungal target whose inhibition compromises ergosterol biosynthesis; and bovine xanthine oxidase (3NVY) as an oxidative-stress-related target, selected as a structurally conserved proxy for antioxidant activity given the high sequence and active-site homology with the human enzyme and its widespread use in polyphenol docking studies. Together, these four receptors provide a coherent ensemble for relating the dominant phenolic constituents of *D. viscosa* to its observed antioxidant, antibacterial, and antifungal activities. It should be noted that the phytochemical ligands exhibited substantially lower affinity for 5TZ1 (best score: −10.0 kcal/mol) than the co-crystallized antifungal VT1161 (−12.7 kcal/mol), reflecting the superior geometric complementarity of the synthetic drug to its native binding pocket; these computed scores therefore serve as a relative ranking tool for the plant-derived compounds rather than as absolute potency predictors.

**Figure 7:**
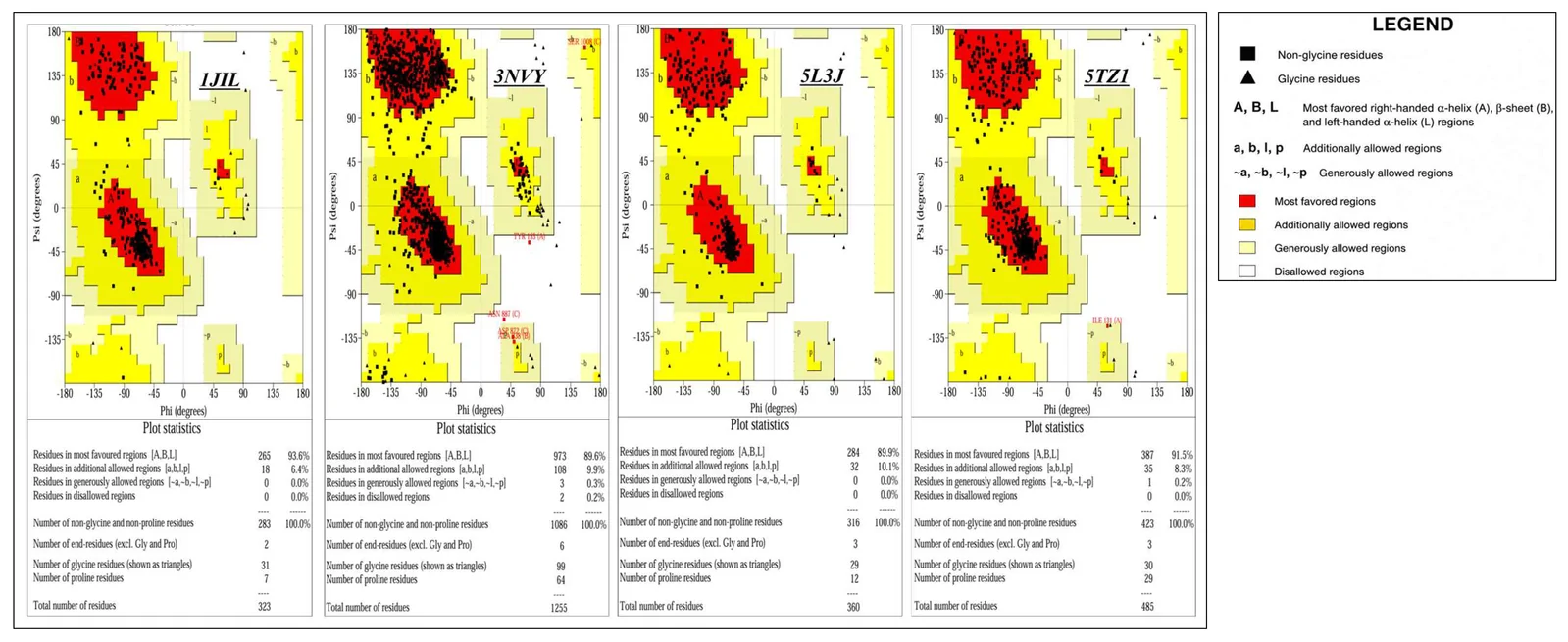
Ramachandran plot validation of the protein structures selected for molecular docking (1JIL, 3NVY, 5L3J, and 5TZ1).

Ramachandran plot analysis confirmed the stereochemical quality of all four receptors: 1JIL (93.6% favoured), 5TZ1 (91.5%), 3NVY (89.6%), and 5L3J (89.9% favoured, 10.1% additionally allowed). No disallowed residues were found in 1JIL, 5TZ1, or 5L3J, and only 0.2% in 3NVY, confirming the structural soundness of all selected protein models (Figure 7).

The affinity ranking summarized in Table 6 revealed a distinctly target-dependent interaction pattern. For 1JIL, kaempferol-3-O-pentoside displayed the most favorable binding energy (−10.3 kcal/mol), slightly surpassing the co-crystallized reference ligand (−9.7 kcal/mol), while quercetin-O-glucuronide and quercetin-O-hexoside also scored strongly (−10.1 kcal/mol). For 3NVY, di-O-caffeoylquinic acid afforded the best phytochemical score (−8.2 kcal/mol), followed by caffeoylquinic acid and kaempferol-7-O-hexoside (−7.9 and −7.6 kcal/mol, respectively), although the native ligand remained marginally more favorable (−8.6 kcal/mol). In the case of 5L3J, di-O-caffeoylquinic acid again emerged as the best-ranked compound (−7.9 kcal/mol), outperforming the co-crystallized inhibitor (−7.0 kcal/mol), with caffeoylquinic acid, kaempferol-7-O-hexoside, and kaempferol-3-O-pentoside clustering closely behind. For 5TZ1, kaempferol-7-O-hexoside was clearly dominant among the plant-derived ligands (−10.0 kcal/mol), followed by caffeoylquinic acid (−9.4 kcal/mol) and di-O-caffeoylquinic acid (−9.1 kcal/mol), although the native VT1161-bound complex remained energetically superior (−12.7 kcal/mol). Overall, the docking matrix indicates that glycosylated flavonols and dicaffeoylquinic acid derivatives are the principal contributors to target engagement, likely because they combine aromatic scaffolds capable of pi-driven recognition with dense oxygenation patterns that promote multipoint hydrogen bonding inside polar enzymatic pockets. These computed energies should not be interpreted as direct potency values or in vitro activity predictors, but they do provide a coherent mechanistic framework for prioritizing kaempferol-3-O-pentoside, di-O-caffeoylquinic acid, and kaempferol-7-O-hexoside as the most plausible bioactive constituents of *D. viscosa* for subsequent experimental validation.

**Figure 8:**
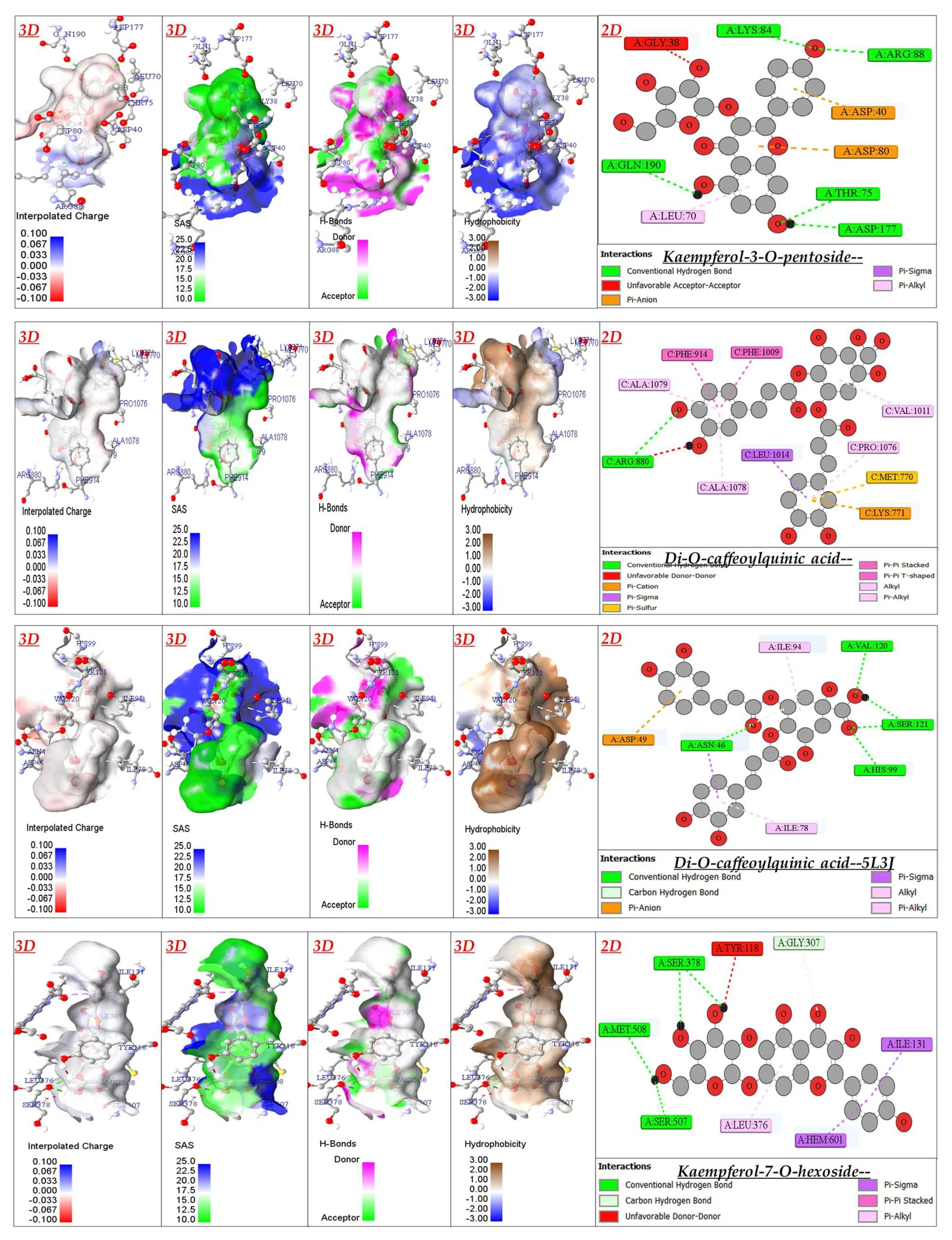
Combined 2D interaction diagrams and 3D surface analyses of the top-ranked docked complexes. Electrostatic, solvent-accessible surface (SAS), hydrogen-bond donor/acceptor, and hydrophobicity maps are shown for kaempferol-3-O-pentoside-1JIL, di-O-caffeoylquinic acid-3NVY, di-O-caffeoylquinic acid-5L3J, and kaempferol-7-O-hexoside-5TZ1 (top to bottom).

To rationalize the strongest scores at the structural level, the top-ranked ligand-receptor complex for each target was further examined by combined 2D interaction mapping and 3D surface analyses of electrostatics, solvent-accessible surface (SAS), hydrogen-bond potential, and hydrophobicity (Figure 8).

**Figure 9:**
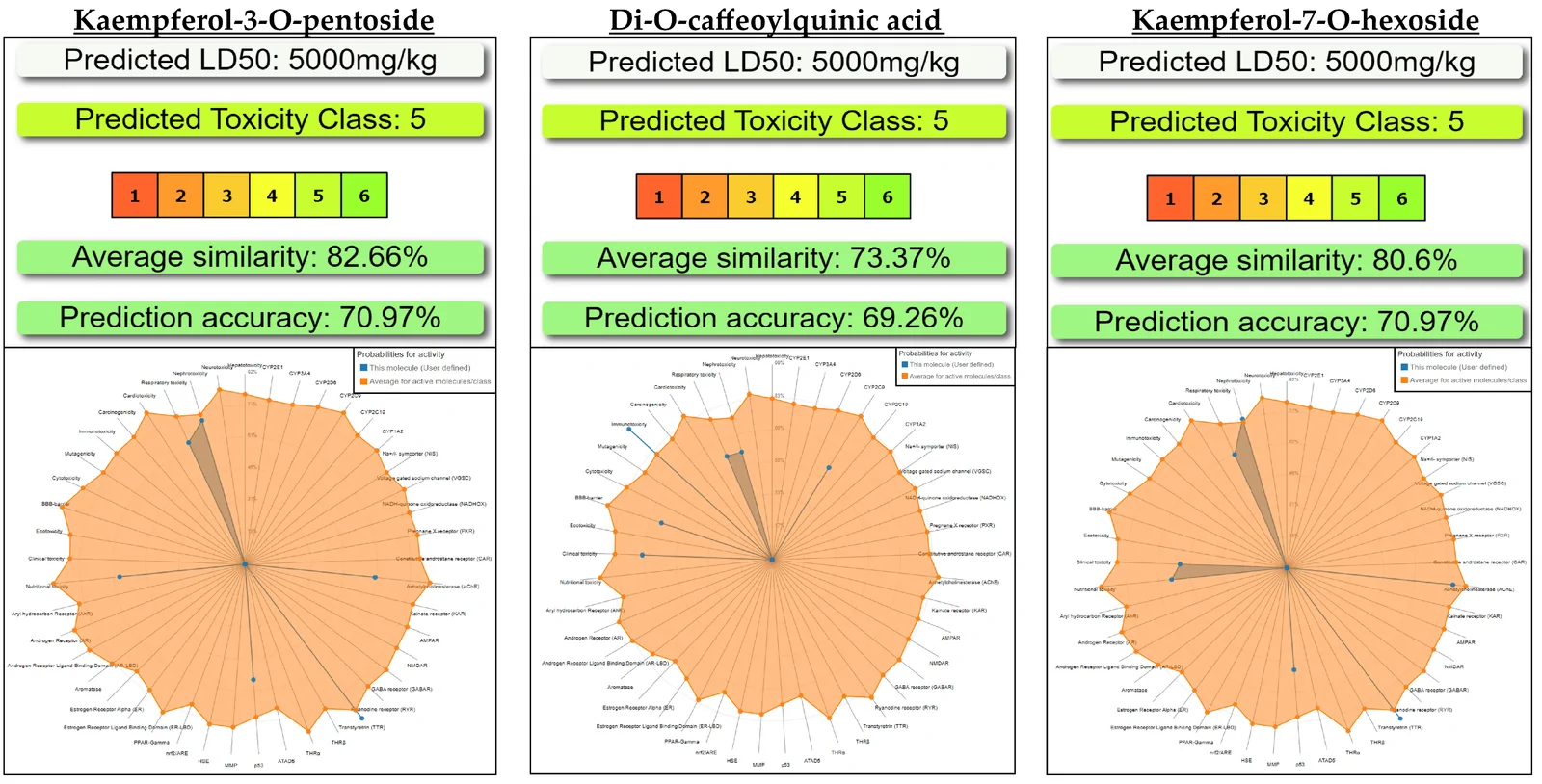
ProTox-3-based in silico toxicity assessment of the top-ranked docked compounds from *Dittrichia viscosa*: predicted oral LD_50_, toxicity class, model confidence parameters, and endpoint-specific toxicity radar plots for kaempferol-3-O-pentoside, di-O-caffeoylquinic acid, and kaempferol-7-O-hexoside.

Figure 9 provides a structural rationale for the scores in Table 6. In 1JIL, kaempferol-3-O-pentoside is deeply seated within a polar catalytic pocket, forming a dense hydrogen-bonding network with Lys84, Arg88, Thr75, Asp177, and a glutamine residue, supplemented by π-anion contacts with Asp40 and Asp80 and a hydrophobic contact with Leu70; the balanced burial and multipoint anchoring explain its superior score [49,50,51]. In 3NVY, di-O-caffeoylquinic acid occupies an elongated cavity stabilized by a principal hydrogen-bond anchor at Arg880 and extensive aromatic/hydrophobic contacts with Phe914, Phe1009, and Leu1014; a minor donor-donor conflict rationalizes why it remains slightly less favorable than the co-crystallized ligand [52,53]. In 5L3J, the same ligand spans the ATP-binding cleft, secured by hydrogen bonds with Val120, Ser121, and His99, and hydrophobic contacts with Ile78 and Ile94, with the polar quinic hydroxyls and ester carbonyls making strong donor/acceptor complementarity [54,55]. In 5TZ1, kaempferol-7-O-hexoside aligns within the sterol-binding channel, with the glycosylated portion forming hydrogen bonds with Ser378, Ser507, and Met508, and the flavonol nucleus stabilized by π-type and hydrophobic contacts with the heme environment and Leu376 and Ile131 [56,57]. Across all four complexes, a recurrent combination of geometric fit, multipoint hydrogen bonding, selective hydrophobic enclosure, and π-mediated contacts provides the structural basis for the docking trends in Table 6.

Overall, the molecular docking results consistently identified kaempferol-3-O-pentoside, di-O-caffeoylquinic acid, and kaempferol-7-O-hexoside as the highest-ranked phenolic constituents across the selected biological targets. Although these computational findings do not establish these compounds as the definitive active principles responsible for the biological activities of the crude extract, they identify them as the most promising candidate lead compounds. These results therefore provide a rational basis for prioritizing these molecules in future bioassay-guided fractionation, enzyme inhibition studies, and in vivo pharmacological investigations.

### 2.9. In Silico Toxicity Prediction

To complement the docking results, the three top-ranked phytochemicals (kaempferol-3-O-pentoside, di-O-caffeoylquinic acid, and kaempferol-7-O-hexoside) were analyzed using ProTox-3. All three shared a predicted oral LD_50_ of 5000 mg/kg and were assigned to toxicity class 5, indicating low predicted acute oral toxicity [58,59]. Endpoint-specific radar plots revealed that di-O-caffeoylquinic acid carries the most concerning profile, with strong immunotoxicity and additional alerts for nephrotoxicity, respiratory effects, and CYP2C9 metabolic interaction; the two kaempferol glycosides showed comparatively more limited profiles, though persistent renal and respiratory alerts still warrant targeted experimental follow-up. These results support the biological relevance of the selected hits while highlighting the need for in vivo safety validation (Figure 9).

### 2.10. Limitations

Several limitations of this study should be acknowledged. First, phenolic compound annotation was performed exclusively by HPLC-PDA-ESI-MS comparison with literature data, without the use of authentic analytical standards; all assignments are therefore putative and should be confirmed by co-injection with reference compounds or NMR spectroscopy in future work. Second, the membrane integrity assay relies on OD_260_ measurement of total macromolecule leakage, which cannot distinguish between nucleic acid, protein, or other UV-absorbing intracellular components; complementary methods such as propidium iodide staining or electrolyte release would provide more mechanistically specific evidence. Third, molecular docking scores reflect in silico binding affinities under idealized conditions and do not account for solubility, bioavailability, or metabolic stability; these computed rankings require experimental validation (e.g., enzyme inhibition assays). Fourth, bovine xanthine oxidase (3NVY) was used as a structural proxy for the human enzyme in docking; while sequence and active-site homology support this choice, species-specific differences may influence the ranking of ligands. Fifth, all biological assays were conducted with reference ATCC strains under laboratory conditions; activity against clinical isolates or in vivo models remains to be established. Sixth, phenolic profiling relied on a single reversed-phase gradient with photodiode-array (UV) detection coupled to negative-mode ESI-MS, a configuration optimized for chromophore-bearing phenolic constituents; metabolites that absorb weakly in the UV range or ionize poorly under these conditions—such as certain sugars, aliphatic organic acids, or small peptides—may therefore be under-represented, and alternative elution programs or complementary detectors (e.g., evaporative light-scattering or charged-aerosol detection) could resolve additional constituents in future work. These limitations do not invalidate the findings but define the scope of conclusions that can be drawn and highlight priority directions for follow-up research.

## 3. Materials and Methods

### 3.1. Plant Material

Leaves of *Dittrichia viscosa* were collected from the Rmilate Forest in Tangier, northern Morocco (35.7876° N, 5.8653° W). Botanical identification was performed by Mohamed El Kadiri (Faculty of Sciences, Tetouan, Morocco), and a voucher specimen was deposited under the reference number LAMSE-DV-22-002. After collection, the leaves were separated from stems and extraneous plant material, then evenly spread in a laboratory ventilated incubator and dried at 40 °C under dark conditions to minimize fungal growth and preserve phytochemical integrity. Following a drying period of two weeks, the dried leaves were finely ground into powder using a laboratory grinder. The resulting powder was stored at room temperature in airtight containers, protected from light, until further analyses.

### 3.2. Preparation of Extracts

#### Extraction of Phenolic Compounds

Dried leaves of *Dittrichia viscosa* (4.5 g) were subjected to exhaustive extraction for 24 h under continuous magnetic stirring using 80% (*v*/*v*) methanol–water (45 mL), prepared from methanol (Sigma-Aldrich, St. Louis, MO, USA) and distilled water, as extraction solvents. The resulting extracts were successively filtered through Whatman No. 1 filter paper (Cytiva, Marlborough, MA, USA) and a 0.45 µm syringe membrane filter (MilliporeSigma, Burlington, MA, USA) to remove particulate matter. Solvent evaporation was carried out at 37 °C under controlled conditions to obtain concentrated extracts, which were then stored at 4 °C in the dark until further analyses.

An aqueous methanol solution (80%, *v*/*v*) was selected based on previous studies demonstrating that hydroalcoholic mixtures containing 70–80% methanol provides high extraction efficiency for phenolic acids, flavonoids, and tannins. The presence of water enhances the swelling of plant tissues and facilitates solvent penetration, whereas methanol efficiently solubilizes moderately polar phenolic compounds, thereby improving the overall recovery of bioactive constituents.

### 3.3. Elemental Trace Analysis

#### Inductively Coupled Plasma-Atomic Emission Spectroscopy (ICP-AES)

Approximately 0.1 g of powdered *Dittrichia viscosa* leaves was digested with 3 mL of concentrated HCl (37%) and 1 mL of concentrated HNO_3_ (65%). The mixture was left at room temperature for 24 h for predigestion, followed by heating at 95 °C for 2 h to ensure complete mineralization. After cooling, the digest was diluted to 25 mL with ultrapure water and filtered through a 0.45 µm membrane filter [60].

Elemental analysis was performed using an ICP ULTIMA EXPERT spectrometer (HORIBA, Palaiseau, France) under standard operating conditions, with analytical wavelengths selected according to the manufacturer’s recommendations.

### 3.4. Phytochemical Screening

#### 3.4.1. Total Phenol Content (TPC)

Total phenolic content was determined using the Folin–Ciocalteu method, following [61] with minor modifications. Briefly, 100 µL of extract solution (1 mg/mL) was mixed with 400 µL of Folin–Ciocalteu reagent (1:10, *v*/*v*), followed by the addition of 1 mL of Na_2_CO_3_ (7%, *m*/*v*). Distilled water was added to obtain a final volume of 1.6 mL, and the mixture was incubated in the dark at room temperature for 30 min. Absorbance was measured at 765 nm using a spectrophotometer. Gallic acid was used for calibration, and results were expressed as mg gallic acid equivalents (GAE) per g of dry extract.

#### 3.4.2. Total Flavonoid Content (TFC)

Total flavonoid content was determined according to the method of [62]. Briefly, 200 µL of extract was mixed with potassium acetate (1 M), aluminum chloride (10%), methanol (50%), and distilled water. The reaction mixture was incubated in the dark at room temperature for 30 min, and absorbance was measured at 415 nm using a spectrophotometer. Quercetin was used as the calibration standard, and results were expressed as mg quercetin equivalents (QE) per g of dry extract.

#### 3.4.3. Total Tannin Content (TTC)

Total tannin content was determined using the Folin–Ciocalteu method according to [60], with minor modifications. Briefly, 100 µL of extract was mixed with 1 mL of sodium carbonate solution (30%) and 0.5 mL of Folin–Ciocalteu reagent. The mixture was incubated in the dark at room temperature for 30 min, and absorbance was measured at 700 nm. Tannic acid was used to construct the calibration curve, and results were expressed as mg tannic acid equivalents (TAE) per g of dry extract.

### 3.5. Fourier Transform Infrared Spectroscopy Analysis (FTIR)

FTIR spectroscopy was performed to identify the functional groups present in the methanolic extract of *Dittrichia viscosa*. The extract was mixed with potassium bromide (KBr) and compressed into a pellet prior to analysis. FTIR spectra were recorded using an FTIR spectrophotometer in the range of 4000–400 cm^−1^, with a spectral resolution of 4 cm^−1^ and 32 co-added scans.

### 3.6. Analysis of Phenolic Compounds Using HPLC-PDA-ESI-MS

Phenolic profiling of *Dittrichia viscosa* leaf extracts was performed using an HPLC-UV-ESI-MS system consisting of an Ultimate 3000 HPLC (Dionex, Thermo Scientific, Waltham, MA, USA) coupled to a Q-Exactive Plus high-resolution mass spectrometer (Thermo Scientific, Waltham, MA, USA). Chromatographic separation was achieved on a BDS Hypersil C18 column (150 × 4.6 mm, 5 μm) using a binary mobile phase system composed of water (A) and acetonitrile (B), both acidified with 0.1% formic acid.

The gradient elution program was as follows: 2% B (0 min), 25% B (15 min), 45% B (30 min), 95% B (40 min), and re-equilibration to 2% B (43 min). The flow rate was set at 0.2 mL/min, with an injection volume of 10 μL and a total run time of 55 min, followed by a 10 min post-run. UV detection was monitored over the range 190–700 nm.

Mass spectrometric analysis was conducted in negative electrospray ionization mode (ESI−) over an *m*/*z* range of 40–1500, with MS/MS spectra acquired at collision energies of 10, 20, and 30 eV. Phenolic compounds were putatively identified based on retention time, UV spectra, accurate mass measurements, and fragmentation patterns, in comparison with literature data. No external analytical standards were used, and relative peak intensities were employed to estimate the abundance of detected metabolites.

### 3.7. Antioxidant Activity Assessment

#### 3.7.1. Radical Scavenging Assay Using DPPH

The free radical scavenging activity of the extracts was evaluated using the DPPH assay, following a previously described method [63]. Extracts were tested at concentrations ranging from 1 to 0.015625 mg/mL. Briefly, 750 µL of DPPH solution prepared in methanol was mixed with 250 µL of each extract. After incubation in the dark at room temperature for 30 min, absorbance was measured at 517 nm using a UV–visible spectrophotometer. The antiradical activity was expressed as IC_50_ values, corresponding to the concentration required to scavenge 50% of DPPH radicals.

#### 3.7.2. Radical Scavenging Assay Using ABTS

The ABTS radical scavenging activity was evaluated following the method of [64], with minor modifications. The ABTS•^+^ solution was generated by reacting 7 mM ABTS with 2.45 mM potassium persulfate and incubating the mixture at 30 °C until an absorbance of 0.72 at 734 nm was obtained. For the assay, 925 µL of the ABTS•^+^ solution was mixed with 75 µL of extract at concentrations ranging from 1 to 0.015625 mg/mL. Absorbance was recorded at 734 nm, and results were expressed as IC_50_ values, representing the concentration required to scavenge 50% of ABTS radicals.

#### 3.7.3. Ferric Reducing Power Assay (FRAP)

The ferric reducing antioxidant power (FRAP) of the extracts was determined according to the method of [65]. The FRAP reagent was freshly prepared by mixing TPTZ, FeCl_3_, and acetate buffer in a 10:1:1 (*v*/*v*/*v*) ratio. Diluted extracts were added to the FRAP reagent and incubated at 30 °C, and absorbance was measured at 593 nm. Trolox was used to generate the calibration curve, and results were expressed as mg Trolox equivalents (TE) per g of dry extract.

#### 3.7.4. Oxygen Radical Absorbance Capacity Assay (ORAC)

The ORAC assay was performed according to [66,67] using a 96-well microplate reader thermostated at 37 °C. Briefly, 120 µL of fluorescein solution (80 nM) prepared in phosphate-buffered saline was added to each well containing the extract. Initial fluorescence was recorded, followed by the addition of 2,2′-azobis(2-amidinopropane) dihydrochloride (AAPH) as the peroxyl radical generator. Fluorescence decay was monitored for 120 min at an excitation wavelength of 485 nm and an emission wavelength of 520 nm. ORAC values were calculated using the method described by [68] with Gen5 2.0 software (BioTek Instruments, Winooski, VT, USA), using Trolox as the calibration standard, and results were expressed as mg Trolox equivalents (TE) per g of extract.

### 3.8. Antibacterial Activity Assessment

The antibacterial activity of *Dittrichia viscosa* leaf extracts was evaluated using the agar well diffusion method, following a standardized procedure with minor modifications [69]. Four reference bacterial strains were tested, including two Gram-positive bacteria (*Staphylococcus aureus* ATCC 25923 and *Bacillus subtilis* ATCC 6633) and two Gram-negative bacteria (*Escherichia coli* ATCC 25922 and *Pseudomonas aeruginosa* ATCC 27853). Bacterial suspensions were prepared in sterile saline and adjusted to the 0.5 McFarland standard (≈10^8^ CFU/mL). Mueller–Hinton agar plates were uniformly inoculated by surface swabbing, after which 6 mm diameter wells were aseptically punched into the agar. Each well was filled with 25 µL of extract solution (100 mg/mL). Gentamicin (10 µg/mL) was used as the positive control, while the corresponding extraction solvent served as the negative control. Plates were incubated at 37 °C for 24 h, and antibacterial activity was assessed by measuring the diameter of inhibition zones (mm) surrounding each well. All experiments were performed in triplicate.

#### 3.8.1. Minimum Inhibitory and Bactericidal Concentrations (MIC/MBC)

The minimum inhibitory concentration (MIC) and minimum bactericidal concentration (MBC) of *Dittrichia viscosa* leaf extracts were determined by the broth microdilution method in Mueller–Hinton broth, following CLSI guidelines [70]. Two-fold serial dilutions of the extracts (0.03125–8 mg/mL) were prepared, and bacterial suspensions of *Staphylococcus aureus* ATCC 25923, *Bacillus subtilis* ATCC 6633, *Escherichia coli* ATCC 25922, and *Pseudomonas aeruginosa* ATCC 27853 were adjusted to ≈1 × 10^6^ CFU/mL [71]. After incubation at 37 °C for 24 h, MIC values were defined as the lowest concentration with no visible growth. For MBC determination, aliquots from growth-free wells were subcultured on Mueller–Hinton agar, and the lowest concentration yielding no colonies after 24 h was recorded as the MBC.

#### 3.8.2. Time–Kill Kinetics

Time–kill assays were performed by exposing bacterial suspensions (≈1 × 10^6^ CFU/mL) to extract concentrations of 1× MIC and 2× MIC in Mueller–Hinton broth at 37 °C, following CLSI guidelines [70]. Samples were collected at 0, 2, 4, 6, 8, and 24 h, serially diluted, plated, and incubated for colony counting. Viable counts were expressed as log_10_ CFU/mL, and bactericidal activity was defined as a reduction of ≥3 log_10_ CFU/mL.

#### 3.8.3. Membrane Integrity Assay

Bacterial membrane integrity was evaluated by measuring the release of intracellular components [72]. Mid-log-phase bacterial cells were treated with extract concentrations equivalent to 1× MIC and incubated at 37 °C for 4 h. After centrifugation, the absorbance of the supernatant was measured at 260 nm. An increase in absorbance relative to untreated controls indicated membrane disruption.

### 3.9. Antifungal Activity

The antifungal activity of *Dittrichia viscosa* leaf extracts was evaluated using the direct contact method as described by [73], with minor modifications. Four fungal strains were tested, including dermatophytic fungi (*Trichophyton rubrum* and *Microsporum canis*) and phytopathogenic fungi (*Botrytis cinerea* and *Fusarium oxysporum*). Briefly, 500 µL of each extract at the tested concentration was incorporated into 20 mL of molten potato dextrose agar (PDA) medium, thoroughly homogenized, and poured into sterile Petri dishes. After solidification, a 6 mm diameter mycelial plug taken from the actively growing margin of a 5-day-old fungal culture was aseptically placed at the center of each plate. Control plates containing PDA without extract were prepared in parallel. All plates were incubated at 25 ± 2 °C for 5 days, after which fungal growth diameters were measured in two perpendicular directions. Antifungal activity was expressed as percentage inhibition of mycelial growth relative to the control. All assays were performed in triplicate.

### 3.10. Molecular Docking Studies

Molecular docking was carried out using AutoDock Vina v1.2.0 implemented in PyRx v0.9. The crystallographic structures of *Staphylococcus aureus* tyrosyl-tRNA synthetase (1JIL), bovine xanthine oxidase (3NVY), *Escherichia coli* DNA gyrase B (5L3J), and *Candida albicans* sterol 14-alpha-demethylase (5TZ1) were retrieved from the Protein Data Bank and prepared in AutoDockTools v1.5.7 by removing crystallographic water molecules and non-essential heteroatoms, adding polar hydrogens, assigning receptor charges, and converting the macromolecules into PDBQT format [74,75,76,77,78]. The co-crystallized ligands were extracted from their native complexes for redocking-based validation, while structurally relevant receptor features inherent to the catalytic environment were retained where required in order to preserve native pocket architecture. The phenolic constituents tentatively annotated by HPLC-PDA-ESI-MS were geometry-optimized prior to docking, and all permissible torsions were defined before conversion to ligand PDBQT files. Molecular visualization, superposition analysis, and 2D/3D interaction mapping were performed using PyMOL v2.5 and BIOVIA Discovery Studio 2024 [79].

**Figure 10:**
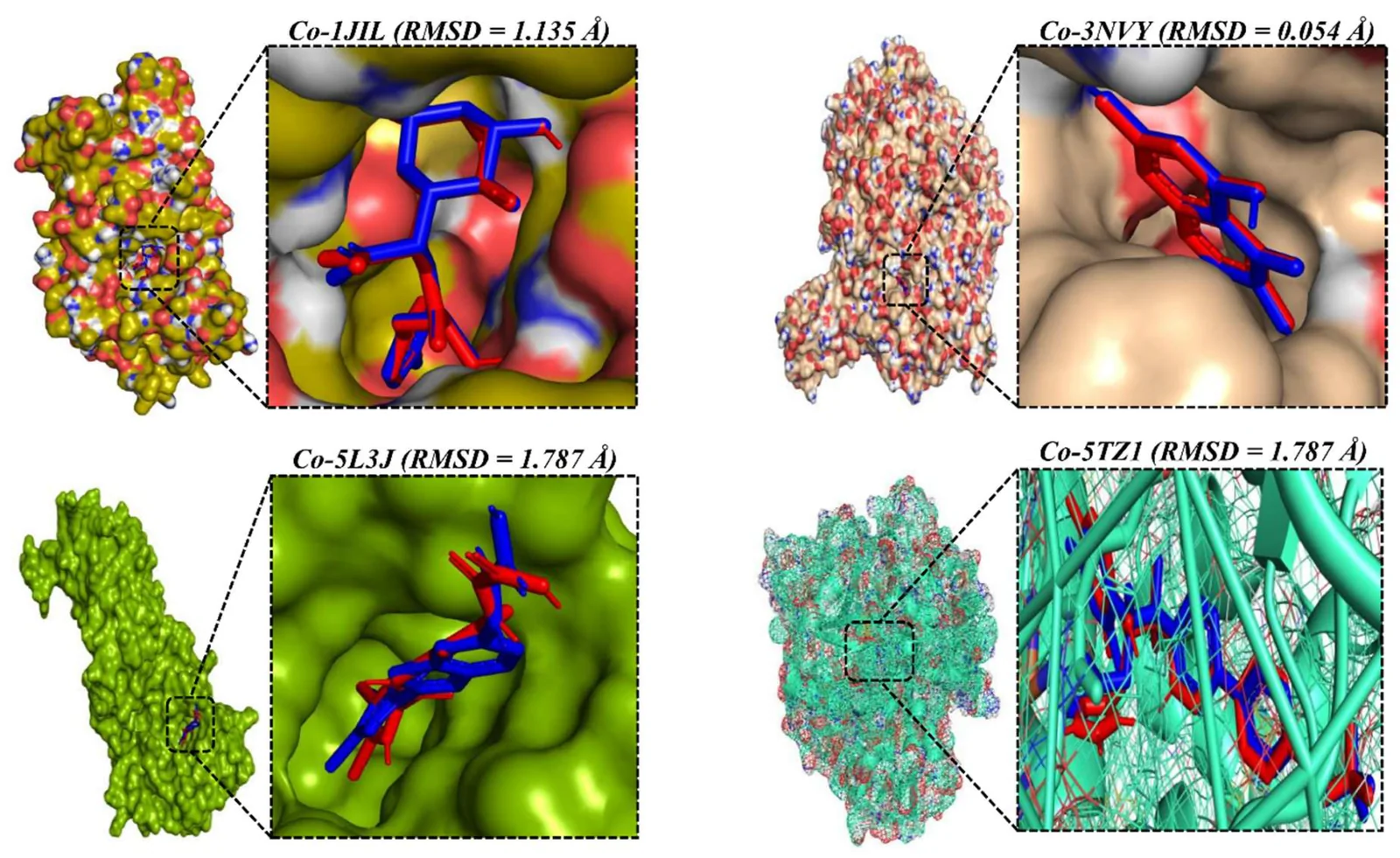
Redocking-based validation of the docking protocol for 1JIL, 3NVY, 5L3J, and 5TZ1. Superposition of the co-crystallized and re-docked ligands within the crystallographic binding sites shows close recovery of the experimental poses, with RMSD values of 1.135, 0.054, 1.787, and 1.787 A, respectively.

Docking reliability was evaluated by re-docking the native ligands into their corresponding crystallographic binding sites using an exhaustiveness value of 10. For 1JIL, the docking box was centered at x = 31.05, y = 11.44, and z = 83.90, with dimensions of 21.91 × 18.01 × 18.94 A, yielding a redocking RMSD of 1.135 A. For 3NVY, the grid center was set at x = 39.21, y = 21.78, and z = 20.00, with dimensions of 20.70 × 17.06 × 17.39 A, affording an RMSD of 0.054 A. For 5L3J, the grid was centered at x = −13.10, y = 19.17, and z = 22.54, with dimensions of 20.03 × 21.52 × 23.93 A, and reproduced the experimental pose with an RMSD of 1.787 A. For 5TZ1, the validation box was centered at x = 70.65, y = 64.93, and z = 4.00, with dimensions of 21.16 × 20.28 × 20.13 A, also giving an RMSD of 1.787 A. All four re-docked poses satisfied the conventional RMSD acceptance criterion of <2.0 Å, confirming the reliability of the protocol. The close superposition between the crystallographic and re-docked poses for all four complexes (Figure 10) confirmed the suitability of the docking protocol for subsequent affinity ranking and interaction analysis.

### 3.11. Prediction of the Toxicity Analysis (ProTox-3)

Canonical SMILES were submitted to ProTox-III to predict acute oral LD_50_, toxicity class, and major organ-specific toxicity alerts [80].

### 3.12. Statistical Analysis

All experiments were performed in triplicate, and results were expressed as mean ± standard deviation (SD). Statistical analyses were carried out using SPSS software (version 23.0; SPSS Inc., Chicago, IL, USA). Differences among the tested extracts were evaluated using one-way analysis of variance (ANOVA) followed by Tukey’s post hoc test, with statistical significance set at *p* < 0.05.

## 4. Conclusions

This study provides a comprehensive characterization of *Dittrichia viscosa* leaf extracts from northern Morocco and demonstrates a clear relationship between their phenolic composition and biological activities. The methanolic extract, enriched in caffeoylquinic acid derivatives, quercetin glycosides, and kaempferol glycosides, consistently exhibited superior antioxidant, antibacterial, and antifungal activities compared with the aqueous extract. Mechanistic antibacterial assays, including MIC/MBC determination, time–kill kinetics, and membrane integrity analysis, supported a bactericidal mode of action associated with membrane disruption. Molecular docking further prioritized kaempferol-3-O-pentoside, di-O-caffeoylquinic acid, and kaempferol-7-O-hexoside as the most promising candidate bioactive compounds, providing mechanistic support for the experimental findings and identifying these molecules as priority targets for future bioassay-guided isolation and pharmacological validation, while in silico toxicity prediction suggested low acute oral toxicity.

Although the phenolic compounds were tentatively identified and all biological evaluations were performed in vitro, the findings highlight *D. viscosa* as a promising natural source of antioxidant and antimicrobial agents. Future studies should focus on bioassay-guided isolation of the active constituents, structural confirmation using authentic standards, in vivo validation, and pharmacokinetic and safety assessments to further support its pharmaceutical and agrochemical applications.

## Figures and Tables

**Table 1:** Elemental Composition Analysis of *Dittrichia Viscosa* Leaves by ICP-AES.

Elements	Mn	Fe	Mg	Ca	Cu	Al	K	Cr	Zn	Ni
mg/kg	40.5	165.375	2935.25	12,476.75	2.775	140.725	29,699	−	1.36	−

**Table 2:** Phenolic compounds tentatively annotated in the methanolic extract of *Dittrichia viscosa* leaves based on retention time, UV absorption, and high-resolution mass spectrometric data.

Peak Number	Rt (min)	[M-H]^−^/[M+H]^+^ (*m*/*z*)	MS^2^ Ions (*m*/*z*)	Molecular Formula	Proposed Compound	Molecular Weight (g/mol)
1	3.98	179	161, 135	C_9_H_8_O_4_	Caffeic acid	180.16
2	9.03	173	155, 111	C_7_H_10_O_5_	Shikimic acid	174.15
3	16.31	353	191, 179, 173	C_16_H_18_O_9_	Caffeoylquinic acid	354.31
4	23.73	153	109, 108	C_7_H_6_O_4_	Dihydroxybenzoic acid	154.12
5	23.92	477	301, 179	C_21_H_18_O_13_	Quercetin-O-glucuronide	478.36
6	27.87	463	301, 179	C_21_H_20_O_12_	Quercetin-O-hexoside 1	464.38
7	30.73	463	301, 179	C_21_H_20_O_12_	Quercetin-O-hexoside 2	464.38
8	31.73	447	285, 255	C_21_H_20_O_11_	Kaempferol-7-O-hexoside	448.38
9	33.91	417	285, 255	C_20_H_18_O_10_	Kaempferol-3-O-pentoside	418.35
10	36.71	515	353, 191, 179	C_25_H_24_O_12_	di-O-caffeoylquinic acid	516.45
11	37.96	515	353, 191	C_25_H_24_O_12_	iso-di-O caffeoylquinic acid I	516.45
12	38.50	515	353, 191	C_25_H_24_O_12_	iso-di-O-caffeoylquinic acid II	516.45

**Table 3:** Inhibition zone diameters (mm) of aqueous and methanolic leaf extracts of *Dittrichia viscosa* against selected Gram-positive and Gram-negative bacteria. Values are mean ± SD (n = 3). Gentamicin (10 µg/mL) positive control: inhibition zones ranged from 18.0 to 28.5 mm across the four strains. Different superscript letters within columns denote significant differences (*p* < 0.05, Tukey’s test).

Extracts	Inhibition Zone Diameter			
	*E. coli*	*P. aeruginosa*	*B. subtilis*	*S. aureus*
**Aqueous**	10.2 ± 0.6 ^c^	8.4 ± 0.5 ^c^	13.1 ± 0.7 ^c^	11.6 ± 0.6 ^c^
**Methanolic**	16.8 ± 0.8 ^a^	13.5 ± 0.7 ^a^	20.4 ± 0.9 ^a^	18.2 ± 0.8 ^a^

**Table 4:** Minimum inhibitory concentration (MIC), minimum bactericidal concentration (MBC), and MBC/MIC ratios of aqueous and methanolic leaf extracts of *Dittrichia viscosa* against selected bacterial strains.

Extracts	Inhibition Parameters	Bacteria Strains			
		*E. coli*	*P. aeruginosa*	*B. subtilis*	*S. aureus*
**Aqueous**	MIC	2	4	1	2
	MBC	4	8	2	4
	MBC/MIC	2	2	2	2
**Methanolic**	MIC	0.5	1	0.25	0.5
	MBC	1	2	0.5	1
	MBC/MIC	2	2	2	2

**Table 5:** Antifungal activity of aqueous and methanolic leaf extracts of *Dittrichia viscosa* expressed as percentage inhibition of mycelial growth of selected fungal strains.

Extracts	Concentration (mg/mL)	Mycelial Inhibition (%)			
		*Trichophyton rubrum*	*Microsporum canis*	*Botrytis cinerea*	*Fusarium oxysporum*
**Aqueous**	**0.5**	18.0 ± 2.0 ^d^	15.0 ± 2.0 ^d^	10.0 ± 1.0 ^d^	8.0 ± 1.0 ^d^
	**1.0**	32.0 ± 3.0 ^c^	28.0 ± 2.0 ^c^	18.0 ± 2.0 ^c^	15.0 ± 2.0 ^c^
	**2.0**	48.0 ± 3.0 ^b^	44.0 ± 3.0 ^b^	30.0 ± 2.0 ^b^	26.0 ± 2.0 ^b^
	**4.0**	63.0 ± 4.0 ^a^	58.0 ± 3.0 ^a^	42.0 ± 3.0 ^a^	38.0 ± 3.0 ^a^
**Methanolic**	**0.5**	35.0 ± 3.0 ^d^	30.0 ± 2.0 ^d^	22.0 ± 2.0 ^d^	18.0 ± 2.0 ^d^
	**1.0**	58.0 ± 4.0 ^c^	52.0 ± 3.0 ^c^	38.0 ± 3.0 ^c^	32.0 ± 3.0 ^c^
	**2.0**	75.0 ± 4.0 ^b^	70.0 ± 4.0 ^b^	55.0 ± 3.0 ^b^	48.0 ± 3.0 ^b^
	**4.0**	88.0 ± 3.0 ^a^	84.0 ± 3.0 ^a^	68.0 ± 4.0 ^a^	62.0 ± 4.0 ^a^

Values are expressed as mean ± SD (n = 3). Different superscript letters within the same column and extract indicate significant differences (*p* < 0.05) according to Tukey’s post hoc test.

**Table 6:** Docking binding energies (kcal/mol) of selected phenolic compounds and co-crystallized ligands against the validated targets 1JIL, 3NVY, 5L3J, and 5TZ1. N/A: Not Applicable.

Molecules	1JIL	3NVY	5L3J	5TZ1
Caffeic acid	−7.2	−7.6	−6.4	−6.9
Caffeoylquinic acid	−8.8	−7.9	−7.7	−9.4
Di-O-caffeoylquinic acid	−9.9	−8.2	−7.9	−9.1
Quercetin-O-glucuronide	−10.1	−5.8	−7.2	−6.9
Quercetin-O-hexoside	−10.1	−6.3	−7.3	−8.1
Kaempferol-7-O-hexoside	−8.7	−7.6	−7.6	−10.0
Kaempferol-3-O-pentoside	−10.3	−6.2	−7.4	−8.4
Co-1JIL	−9.7	N/A	N/A	N/A
Co-3NVY	N/A	−8.6	N/A	N/A
Co-5L3J	N/A	N/A	−7.0	N/A
Co-5TZ1	N/A	N/A	N/A	−12.7

## Data Availability

The data supporting the findings of this study are available from the corresponding author upon reasonable request.
